# Intranasal Nanoliposomes Delivering Interferon Lambda with Enhanced Mucosal Retention as an Antiviral

**DOI:** 10.34133/bmr.0287

**Published:** 2025-11-21

**Authors:** Seungju Yang, Jeongwon Yun, Jae Hyuk Kwon, Ji Eun Oh, Ho Min Kim, Hyun Jung Chung

**Affiliations:** ^1^ Department of Biological Sciences, Korea Advanced Institute of Science and Technology (KAIST), Daejeon 34141, Republic of Korea.; ^2^ Graduate School of Medical Science and Engineering, Korea Advanced Institute of Science and Technology (KAIST), Daejeon 34141, Republic of Korea.; ^3^ AI Co-Research & Education for Innovative Drug (AI-CRED) Institute, Daejeon 34141, Republic of Korea.

## Abstract

Respiratory virus infections continue to pose a substantial global health challenge, requiring effective prophylactic and therapeutic strategies. Type III interferon (IFN-λ) has shown promise as an antiviral agent that strongly inhibits viral replication while minimizing systemic inflammation. Intranasal administration of IFN-λ allows easy access to the respiratory mucosa, enhancing localized antiviral responses. However, clinical application of IFN-λ is hindered by rapid mucociliary clearance, limited mucosal adhesion, and susceptibility to proteolytic degradation. Here, we develop nanoliposomes that can deliver IFN-λ through an intranasal route (NLp@IFN-λ) and act as an effective antiviral. We demonstrate that the nanoliposomes enable efficient penetration of IFN-λ in a mucus-mimicking model while allowing controlled release of the protein in vitro. NLp@IFN-λ treatment could effectively up-regulate interferon-stimulated genes in A549 cells, without inducing cytotoxicity. Finally, in vivo delivery of NLp@IFN-λ through a nasal route demonstrates prolonged retention and reduces viral load in nasal tissues in an infection model with influenza virus. This study demonstrates the potential of NLp@IFN-λ as an effective nasal delivery platform for prophylaxis of respiratory virus infections.

## Introduction

Viral respiratory infections present a serious challenge in public health, highlighted by the recent COVID-19 pandemic [1,2]. Effective prophylactic and therapeutic strategies are needed to treat the respiratory infections caused by viruses, such as influenza A and SARS-CoV-2. Among the antiviral agents, interferons (IFNs) represent key cytokines that induce antiviral immune responses at broad-spectrum [3,4]. In particular, type III IFNs called IFN lambda (IFN-λ) have been shown to be effective in relieving respiratory infections by influenza, human metapneumovirus, and coronavirus [5–7]. Unlike type I IFNs (IFN-α and IFN-β), IFN-λ specifically acts on their receptor complexes (IFN-λR1 and IL-10Rβ) expressed on mucosal epithelial cells, thereby minimizing systemic inflammatory responses [8,9].

Intranasal delivery provides a noninvasive route for therapeutic agents providing immediate access to IFN-λ receptors, leading to localized immune responses in the nasal epithelium [10–12]. Initial sites of respiratory viral infections are mainly located in the nasal and upper respiratory epithelium [13,14]. Therefore, intranasal delivery of IFN-λ can result in high local concentrations of the protein at the sites of respiratory virus entry and replication, enhancing prophylactic antiviral effects while reducing systemic side effects [15,16]. A recent study has reported that nasal administration of IFN-λ2 can reduce viral replications in the nasal tissue and prevent transmission to the lung [17]. Despite its therapeutic potential, the clinical application of IFN-λ-based therapies faces major challenges due to rapid mucociliary clearance, limited mucosal penetration, enzymatic degradation, and poor stability of the protein [18–21]. Therefore, the development of an effective nasal delivery platform that can enhance the penetration and sustain the effect of the therapeutic agent is necessary to improve the efficacy.

Previous studies have reported nasal drug delivery platforms based on liposomes, polymers, and natural polysaccharides, which can improve the stability, retention, and bioavailability of the drug in the nasal environment [22–25]. In particular, liposomes have been used for condensation, solubilization, or preventing enzymatic degradation of drugs when delivered into the respiratory epithelium, but have shown limitations in mucosal adhesion and penetration [16,26–29]. Synthetic polymers have been demonstrated as efficient drug carriers with controllable release properties, but pose challenges due to limitations in protein stability and loading efficiency, and the severe in vivo toxicity [30–32]. Among the natural polysaccharides, chitosan (CS) has been known for its unique features as a mucoadhesive material with high biocompatibility, offering a promising strategy for intranasal delivery of antiviral drugs [33–35]. Previously, intranasal administration of CS nanoparticles delivering malate dehydrogenase induced mucosal immune response in the upper respiratory tract [36]. Another study reported that CS nanoparticles releasing ovalbumin showed mucoadhesive properties and sustained release for 96 h in the intestinal fluid [37]. However, an effective and robust platform that can overcome rapid mucociliary clearance while maintaining the bioactivity of structurally labile proteins, such as the IFNs, needs to be developed.

**Fig. 1.**
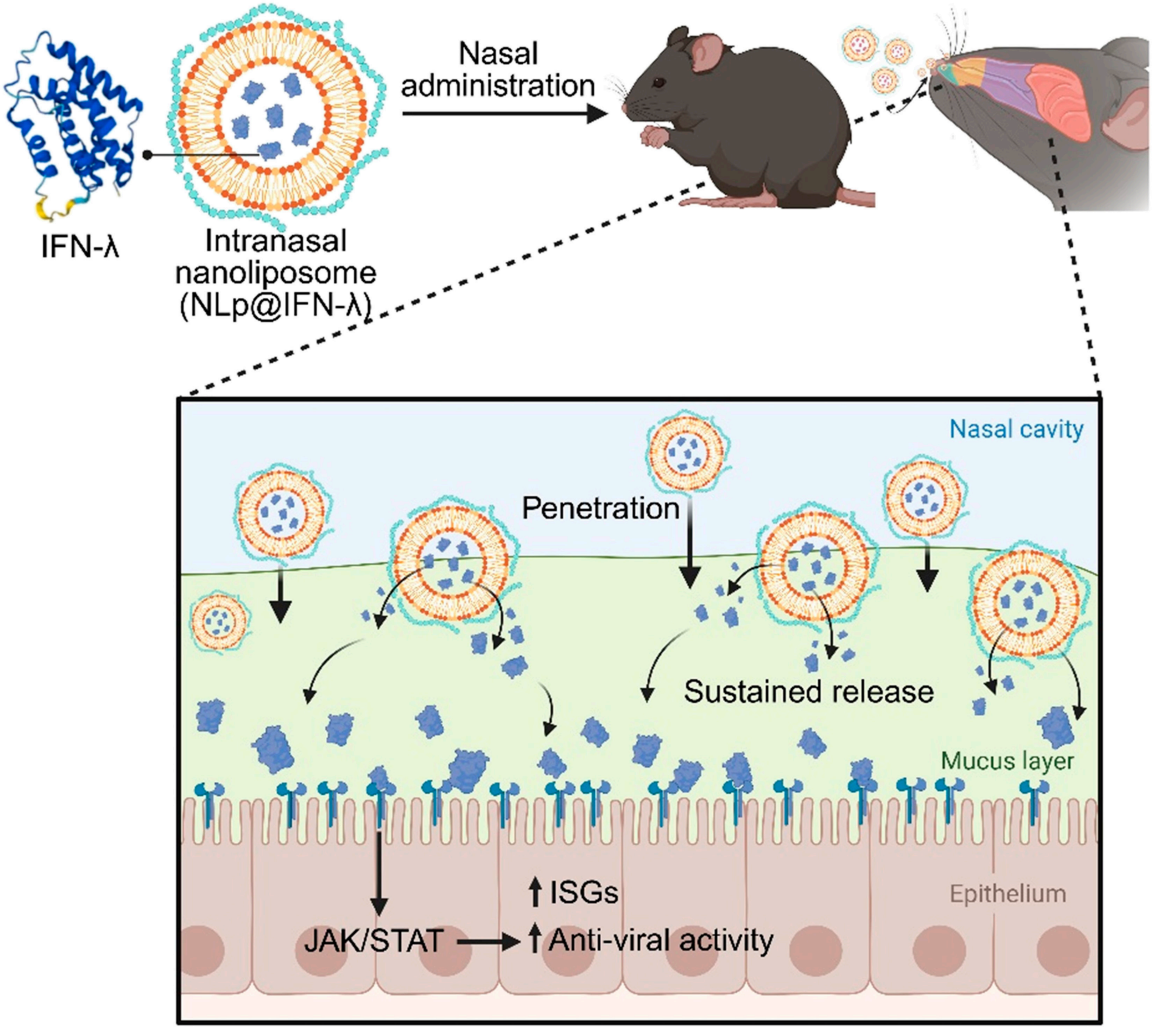
Overall schematic on the development of intranasal nanoliposomes delivering IFN-λ (NLp@IFN-λ) for antiviral therapy. NLp@IFN-λ is nasally administered, resulting in prolonged nasal retention of IFN-λ, leading to up-regulation of ISGs and antiviral effects.

Here, we introduce an intranasal nanoliposome-based platform (NLp@IFN-λ) that can effectively deliver IFN-λ3 through the nasal mucosa and lead to antiviral effects in vivo (Fig. 1). Anionic liposomes encapsulating IFN-λ were prepared, followed by complexation with mucoadhesive polymer to generate NLp@IFN-λ, which showed efficient mucus penetration and sustained release of IFN-λ. Treating NLp@IFN-λ to cells could successfully activate IFN-stimulated genes (ISGs) without causing cytotoxicity in vitro. Furthermore, intranasal delivery of NLp@IFN-λ in vivo resulted in localization and prolonged retention of IFN-λ in the nasal cavity, leading to potent antiviral effects against influenza A virus (IAV). These findings highlight the potential of the study, providing an effective nasal delivery platform for therapeutic proteins, as well as a nasal formulation for prophylactic treatment of respiratory viral infections.

## Materials and Methods

### Materials

Low-molecular-weight CS (LCS, #448869), medium-molecular-weight CS (MCS, #448877), and hydroxyethylcellulose (HEC, #09368) were purchased from Sigma-Aldrich. Alkyne-borondipyrromethene (BODIPY, #CLK-045) was purchased from Jena Bioscience. Alexa Fluor 647-N-hydroxysuccinimide ester (AF647-NHS ester, #A37566) and Alexa Fluor 750-N-hydroxysuccinimide ester (AF750-NHS ester, #A20111) were purchased from Thermo Fisher Scientific. 1,2-Dipalmitoyl-sn-glycero-3-phospho-(1′-rac-glycerol) (DPPG, #840455P) and 1,2-dipalmitoyl-sn-glycero-3-phosphocholine (DPPC, #850355C) were purchased from Avanti Polar Lipids.

### Methods

#### Preparation of intranasal nanoliposomes

Intranasal nanoliposomes carrying IFN lambda 3 (NLp@IFN-λ) were prepared using the thin-film hydration method, followed by adding CS. Briefly, 10 mg/ml of DPPC and DPPG lipids in chloroform were mixed in a glass vial and the solvent was evaporated in nitrogen gas overnight. The dried lipid film was hydrated with 1 mg/ml of IFN-λ in phosphate-buffered saline (PBS) at 45 °C for 1 h under stirring. Using a glass syringe hand extruder, multilamellar vesicle solution was extruded through 200-nm polycarbonate membrane filters. Then, LCS or MCS was dissolved at 10 mg/ml in 1% (v/v) acetic acid, and added dropwise to the liposome solution and stirred at 37 °C for 1 h. Unbound CS and free IFN-λ were removed by centrifugal filtration using Amicon Ultra-0.5 (100 kDa molecular weight cut-off [MWCO]).

#### Encapsulation efficiency

To construct a standard curve for quantification, IFN-λ was prepared (0.5, 1, 2, and 4 μg) and sodium dodecyl sulfate–polyacrylamide gel electrophoresis (SDS-PAGE) analysis was performed. The result of SDS-PAGE gel was stained with Coomassie Brilliant Blue, and band intensities corresponding to IFN-λ were quantified using ImageJ software. Quantified intensity values were used to construct a standard curve. For calculation of encapsulation efficiency of CS-coated liposomes, NLp@IFN-λ was prepared and disrupted by 0.05% Triton X-100 at 4 °C for 50 min. The resultant was purified by Amicon Ultra-0.5 centrifugal filter (3 kDa MWCO) to remove lipid. The purified IFN-λ was analyzed by SDS-PAGE analysis, followed by staining with Coomassie Brilliant Blue, and its amount was quantified by the standard curve. Encapsulation efficiency was calculated using the following formula:

$$\begin{array}{c} \text{Encapsulation efficiency}\;\left(\%\right)=\\ \frac{\left(\text{Amount of}\;\mathrm{IFN}-\lambda \;\text{recovered from}\;\text{lipo@}\mathrm{IFN}-\lambda \right)}{\left(\text{Total amount of}\;\mathrm{IFN}-\lambda \;\text{initially added}\right)}\times 100 \end{array}$$ (1)

#### Cryo-TEM analysis

Nanoliposomes were diluted to a final concentration of 0.2 mg/ml in distilled water (DW). Cryo-transmission electron microscopy (cryo-TEM) imaging of nanoliposomes was performed using a Tecnai G2 Spirit TWIN transmission electron microscope (FEI, Gatan).

#### Cytotoxicity assay

Cell viability was examined using the Cell Counting Kit-8 (CCK-8 assay, Dojindo Molecular Technologies). Cells (1 × 10^4^) were seeded in a 96-well plate, cultured overnight, and treated with the complexes at an atmosphere of 5% CO_2_ at 37 °C for 12 h. Cells were washed with culture media, treated with 10 μl of water-soluble tetrazolium 8, and incubated at 37 °C for 90 min. The absorbance at 450 nm was measured with Infinite M200 PRO (Tecan), and cell viability was determined based on normalization with values for the control (no treatment).

#### RT-qPCR of ISG levels

Starved A549 cells were incubated with 100 ng/ml of IFN-λs for 12 h. Cells were harvested and RNA was extracted using TaKaRa MiniBEST Universal RNA Extraction Kit (#9767A, TaKaRa). Extracted RNA was reverse-transcribed using a T7 RNA polymerase (#M025, New England Biolabs) according to the kit instructions. Next, quantitative polymerase chain reaction (qPCR) was performed using PowerUP SYBR Green Master Mix (#A25741, Applied Biosystems) on a QuantStudio 3 Real-Time PCR (Thermo Fisher Scientific). The RT-qPCR primers are listed in Table S1. Ct values were normalized against GAPDH, and the relative mRNA expression levels compared to untreated samples (PBS) were calculated using the ΔΔCt method (fold expression = 2^–ΔΔCt^).

#### Measurement of IFN-λ release

To construct a standard curve for quantification, IFN-λ labeled with AF647 was prepared (12.5, 25, 50, 100, and 200 ng/ml) and fluorescence intensity was measured with Infinite M200 PRO (Tecan). A standard curve was generated by plotting the fluorescence intensity versus concentration of IFN-λ. For plotting the release profile of liposome encapsulating IFN-λ, NLp@IFN-λ was placed in a dialysis membrane (100 kDa MWCO) in 100 ml of PBS (pH 7.4) at 30 °C under 800 rpm. At predetermined time points, the fluorescence intensity of external PBS solution was measured with Infinite M200 PRO and the amount of released IFN-λ was calculated using the previously plotted standard curve. Payload release was calculated using the following formula:

$$\begin{array}{c} \text{Payload release}\;\left(\%\right)=\\ \frac{\left(\text{Amount of released}\;\mathrm{IFN}-\lambda \right)}{\left(\text{Total amount of}\;\mathrm{IFN}\;\text{initially encapsulated}\right)}\times 100 \end{array}$$ (2)

#### Penetration assay

For preparation of 1.2% HEC solution, 0.6 g of HEC powder was dissolved in 50 ml of DW and stirred at 70 °C. For 3-dimensional (3D) imaging, 150 μl of 1 mM BODIPY was added in 1.2% HEC solution, followed by coating the 24-well culture plate (SPL Life Sciences, #30024). For synthesis of IFN-λ-AF647, IFN-λ was reacted with AF647-NHS ester at a molar ratio of 1:4 at room temperature for 2 h. The unreacted AF647-NHS ester was removed using an Amicon Ultra-0.5 centrifugal filter (3 kDa MWCO). IFN-λ-AF647 (1 μg) was dropped on the 1.2% HEC-coated 24-well culture plate and observed by confocal microscopy (LSM 880, Carl Zeiss). The intensity of IFN-λ at each plane was measured using Zen Blue (Carl Zeiss).

#### Animals

Six- to eight-week-old male C57BL/6J mice were used and housed in a specific-pathogen-free facility at the Korea Advanced Institute of Science and Technology (KAIST). All procedures used in this study complied with the guidelines and protocol (KA2021-004) of the Institutional Animal Care and Use Committee of KAIST.

#### Immunofluorescence imaging

For immunofluorescence staining, mice were perfused via the left ventricle with ice-cold PBS to remove blood, followed by fixation with 2% paraformaldehyde (PFA; #15710, EMS). For cryo-sectioning of the mouse nasal cavity, heads were postfixed in 4% PFA overnight at 4 °C, decalcified in 0.5 M EDTA (#ML005-01, Welgene) for 96 h at 4 °C, and dehydrated by submersion in 30% sucrose (#SUC01, LPS) for 48 h at 4 °C. Samples were embedded in Tissue-Tek® Optimal Cutting Temperature (O.C.T.) compound (#4583, Sakura), frozen, and sectioned at 15 μm thickness. Frozen nasal sections were stained with 4′,6-diamidino-2-phenylindole (DAPI; #D-9542, Sigma-Aldrich) and mounted using Fluoromount-G (#0100-01, SouthernBiotech). For immunostaining, sections were incubated with a primary antibody, AF488-conjugated anti-mouse CD326 (Ep-CAM) (rat monoclonal, 1:300 dilution; #118210, BioLegend). The secondary antibody used was Alexa Fluor 594 AffiniPure rabbit anti-goat IgG (H+L) (1:1,000 dilution, #305-585-045, Jackson ImmunoResearch). Confocal images were acquired using a Zeiss LSM980 microscope with 10× or 20× objectives.

#### Virus infection and efficacy

For intranasal IFN-λs treatment, mice were nasally administered 2 μg of free IFN-λ and NLp@IFN-λ in 10 μl of PBS. For upper respiratory tract infection, mice were nasally inoculated with 1 × 10^4^ plaque-forming units of influenza A/PR/8/34 virus (H1N1, provided by HK Lee, KAIST, South Korea) in an 8-μl volume per nostril under light anesthesia (4% isoflurane in oxygen). At 2 days postinfection, mice were euthanized and washing fluid and tissue lysates from the upper and lower respiratory tracts were collected as follows. The nasal cavity was gently flushed with 600 μl of PBS containing 0.1% bovine serum albumin (BSA) to obtain nasal wash samples, and bronchoalveolar lavage (BAL) fluid was collected by lung lavage with 1 ml of PBS containing 0.1% BSA. Nasal turbinate tissue and lung tissue were homogenized in minimum essential medium containing 0.3% BSA using a Precellys 24 bead-beating homogenizer (#P000669-PR240-A, Bertin Technologies) and lysing kit tubes (#P000911-LYSK0-A.O, Bertin Technologies).

#### Statistical analysis

All statistical analyses were conducted using GraphPad Prism. All assay experiments were performed in triplicates or quadruplicates. Data were presented as mean ± standard deviation or mean ± standard error. *P* values were obtained using one-way analysis of variance. A *P* value of <0.05 was considered statistically significant (*P < 0.05, **P < 0.01, ***P < 0.001).

## Results and Discussion

### Preparation of intranasal nanoliposomes carrying IFN-λ

**Fig. 2.**
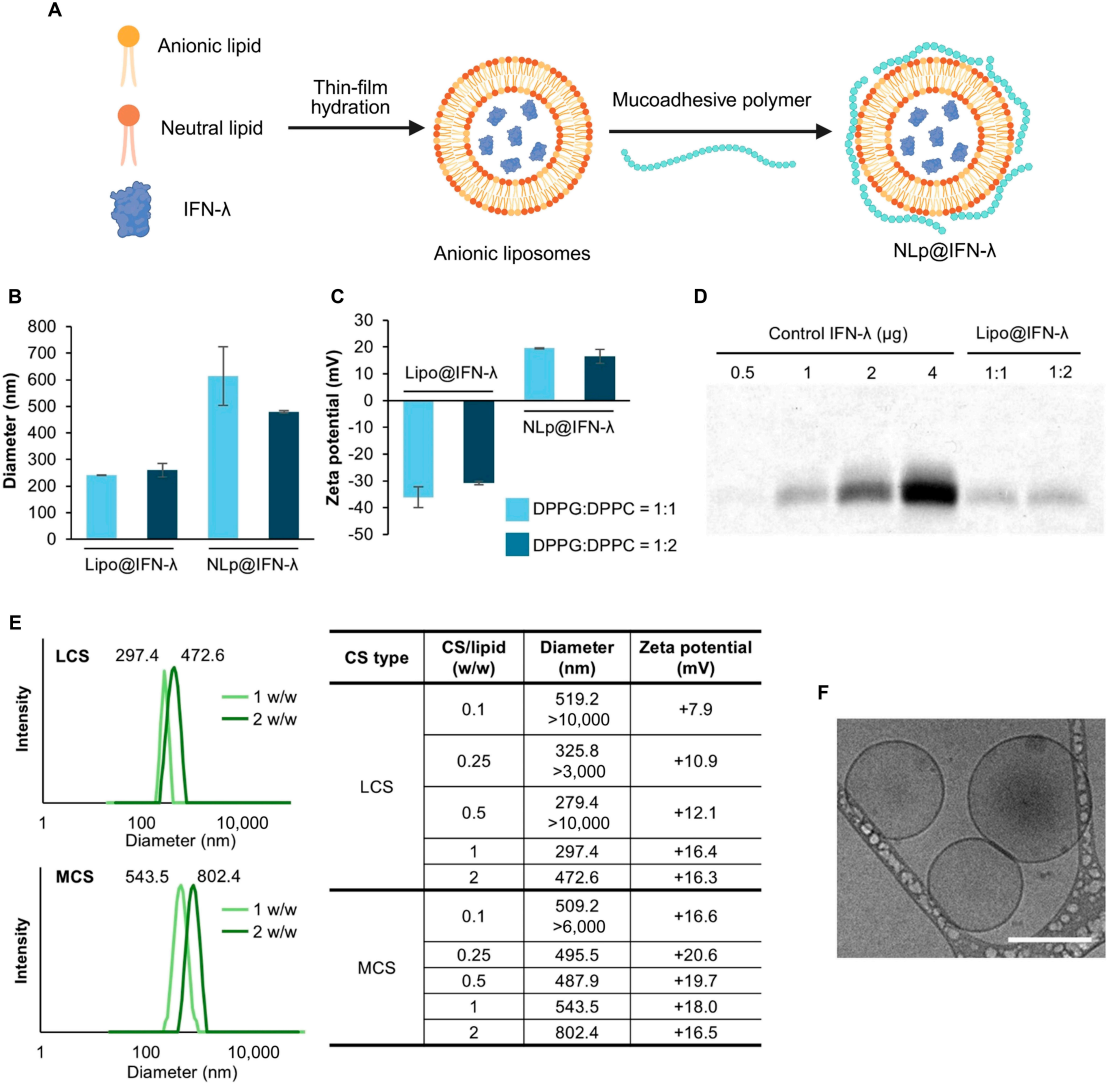
Physicochemical characterization of nanoliposomes carrying IFN-λ. (A) Schematic of preparation of NLp@IFN-λ. (B) Hydrodynamic diameter and (C) zeta potential measurements (*n* = 3) of Lipo@IFN-λ and NLp@IFN-λ (LCS:lipid weight ratio of 2 w/w) prepared at different molar ratios of DPPG and DPPC. (D) SDS-PAGE analysis of IFN-λ retrieved from Lipo@IFN-λ, confirming successful encapsulation. (E) Hydrodynamic diameters and zeta potentials of NLp@IFN-λ using various CS types and CS/lipid weight ratios. (F) Cryo-TEM imaging of NLp@IFN-λ (liposomes composed of DPPG:DPPC = 1:2, prepared with 2 w/w of LCS; scale bar: 200 nm).

As an effective nasal delivery platform for IFN-λ, we developed nanoliposomes that can be retained in the nasal mucosa and exert sustained antiviral activity. Considering that the isoelectric point of IFN-λ is approximately 8.96, we designed anionic liposomes to achieve high encapsulation efficiency by electrostatic interactions between the lipid and the protein (Fig. 2A). First, the molar ratio between the anionic (DPPG) and neutral (DPPC) lipid was varied for optimization using blank liposomes without IFN-λ. Hydrodynamic diameter measurements showed that the sizes of liposomes composed of DPPG and DPPC at molar ratios of 1:1 and 1:2 were approximately 221.0 and 235.1 nm, respectively. Zeta potential analysis revealed highly negative surface charges of −51.0 and −37.8 mV for liposomes composed of DPPG and DPPC at molar ratios of 1:1 and 1:2, respectively, confirming the formation of stable anionic liposomes (Fig. S1). Encapsulation of IFN-λ into the anionic liposomes (Lipo@IFN-λ) resulted in increased particle sizes of 241.9 and 259.9 nm, and slight increases in surface charges to −36.1 and −30.7 mV at DPPG:DPPC ratios of 1:1 and 1:2, respectively (Fig. 2B and C). These results indicate the successful encapsulation of IFN-λ within the anionic liposomes. Next, intranasal nanoliposomes were prepared by adding the mucoadhesive polysaccharide, CS, to the 2 anionic liposomes, to generate NLp@IFN-λ. CS, a polysaccharide derived from chitin, interacts with mucin in the mucus layer by electrostatic interaction, hydrogen bonding, and hydrophobic interaction [33,34]. Hydrodynamic diameters of NLp@IFN-λ appeared to be 613.4 and 479.6 nm for DPPG:DPPC ratios of 1:1 and 1:2, respectively, which were larger compared to anionic liposomes without CS. Zeta potential measurements of NLp@IFN-λ resulted in values of 19.5 and 16.5 mV for DPPG:DPPC ratios of 1:1 and 1:2, respectively. The increase in hydrodynamic diameters and surface charges of NLp@IFN-λ confirmed the successful formation of the nanoliposomes. The encapsulation efficiencies of IFN-λ in Lipo@IFN-λ were 18.7% and 23.9% for DPPG:DPPC ratios of 1:1 and 1:2, respectively (Fig. 2D), demonstrating successful loading of IFN-λ into nanoliposomes. Collectively, we selected liposomes comprising DPPG:DPPC at a molar ratio of 1:2, due to their uniform and optimal size, and high encapsulation efficiency, for further evaluation.

We next examined the influence of the molecular weight of CS on the formation of the nanoliposomes. We used CS with 2 different molecular weights (LCS, 50 to 190 kDa; MCS, 190 to 310 kDa) that were added to the anionic liposomes encapsulating IFN-λ at various weight ratios (Fig. 2E). At LCS weight ratios of below 0.5 w/w and an MCS weight ratio of 0.1 w/w, liposome aggregation was clearly observed. Hydrodynamic diameter measurements showed bimodal size distribution with high polydispersity, suggesting that these conditions were not optimal. In the meantime, increasing the weight ratio to 1 w/w or higher for LCS and above 0.25 w/w for MCS resulted in more stable and monodisperse liposomes. These results demonstrate that controlling the CS-to-lipid ratio and the molecular weight of CS is critical for the successful preparation of the nanoliposomes. Cryo-TEM further demonstrated that NLp@IFN-λ exhibited uniform spherical morphology, confirming the formation of stable and monodisperse nanoliposomes (Fig. 2F and Fig. S2). Taken together, these results demonstrate that nanoliposomes encapsulating IFN-λ were successfully prepared and optimized for further evaluation as a nasal delivery platform.

### Characterization of release and penetration of NLp@IFN-λ

**Fig. 3.**
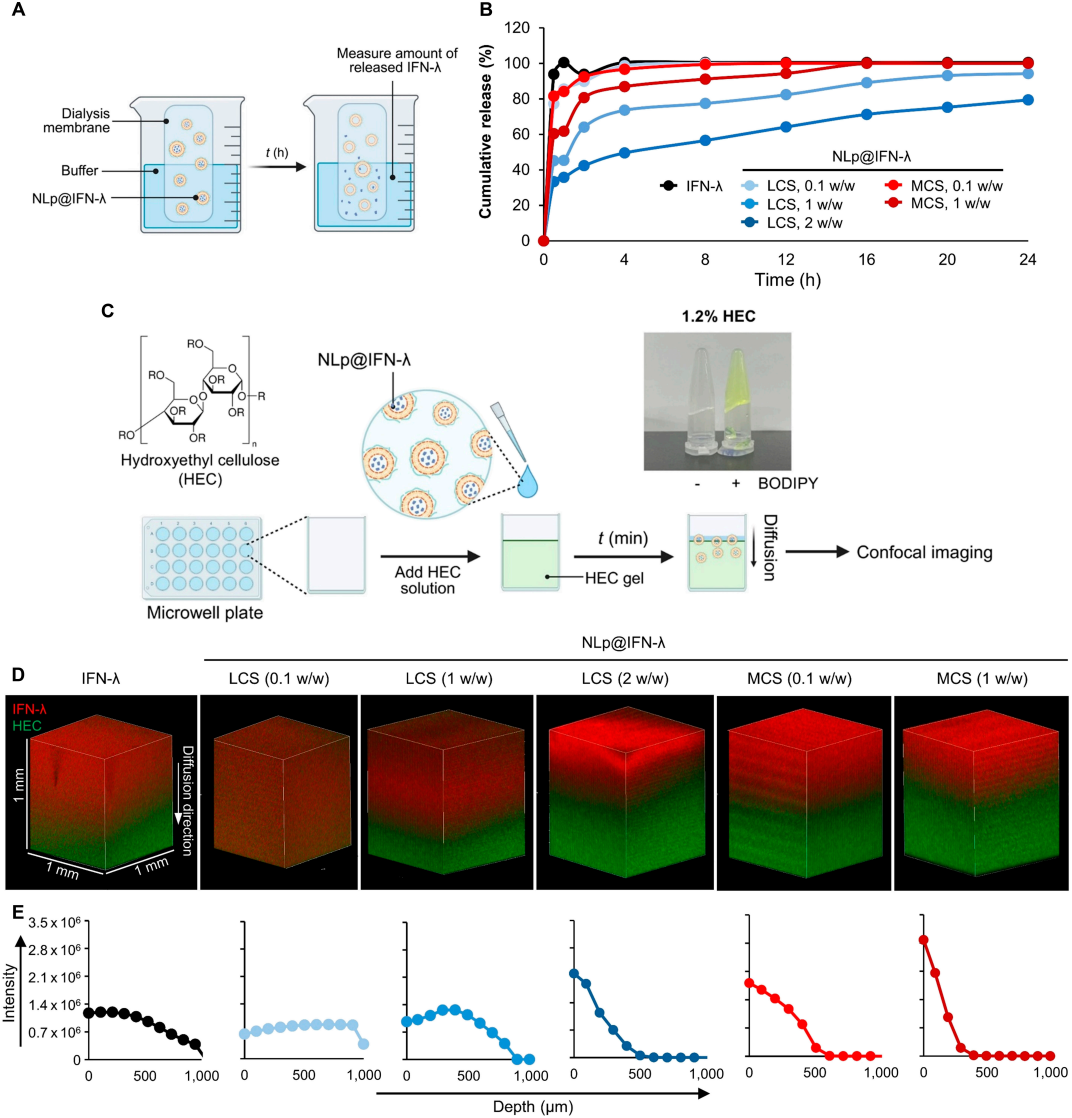
(A) Method for measuring IFN-λ release, and (B) release profiles of IFN-λ from NLp@IFN-λ prepared with various CS molecular weight and weight ratios. (C) Schematic showing the preparation of the HEC gels representing the nasal mucus for penetration studies. Image shows 1.2% HEC gel stained with the BODIPY dye. (D) Confocal microscopy of HEC gels added with NLp@IFN-λ or free IFN-λ, showing diffusion of IFN-λ (red). HEC gels stained with BODIPY (green) for visualization. (E) Quantification of fluorescence intensities along the *z*-axis.

We next examined the release of IFN-λ from the intranasal nanoliposomes prepared with LCS or MCS at various weight ratios (Fig. 3A). The molecular weight and weight ratio of CS in the nanoliposomes were shown to significantly affect the release kinetics of IFN-λ (Fig. 3B). In the case of free IFN-λ, the protein was rapidly released into the buffer solution within 1 h. On the other hand, NLp@IFN-λ prepared with LCS exhibited a release of 85.5%, 45.3%, and 35.7% of the protein for 0.1, 1, and 2 w/w of LCS, respectively, while the ones prepared with MCS showed a release of 84.2% and 61.7% for 0.1 and 1 w/w of MCS, respectively. These results demonstrate that the nanoliposomes prepared with LCS or MCS can release IFN-λ in a sustained manner. After 24 h, the majority of the protein (>99.0%) was released from NLp@IFN-λ prepared with 0.1 w/w LCS, whereas incomplete release was shown at higher ratios (79.4% release for 2 w/w). That is, increasing the CS weight ratio in the nanoliposomes prepared with CS resulted in more sustained release of IFN-λ, which can be due to the presence of a thicker layer of CS. Nanoliposomes prepared with MCS also showed prolonged release of encapsulated IFN-λ, but to a smaller extent compared to the ones prepared with LCS. This can be attributed to the higher efficiency of LCS in surface coverage of the nanoliposomes, leading to more stable and stronger binding of IFN-λ.

To examine the effect of the nanoliposomes on mucus penetration, we prepared a 3D gel model using HEC, to represent the viscous and glycan-rich environment of the native mucus (Fig. 3C). Fluorescence-labeled HEC gels were prepared and the nanoliposomes were applied, followed by 3D imaging analysis by confocal microscopy (Fig. 3D). Treatment of NLp@IFN-λ with 0.1 and 1 w/w of LCS showed substantially enhanced penetration compared to free IFN-λ, suggesting that these formulations can penetrate through the mucus with high efficiency. The nanoliposomes prepared with 2 w/w of LCS and 0.1 to 1 w/w of MCS showed slower penetration, observed by the localization of the IFN-λ signals near the surface of the HEC gel (Fig. 3E). These results can be related to the presence of CS with higher molecular weight or higher weight ratios, which induce excessive mucoadhesion and entrapment of IFN-λ in the nanoliposomes for a longer duration. This is consistent with previous reports showing that CS with a low molecular weight or at smaller doses would lead to efficient mucus penetration, while the ones with a high molecular weight or at larger amounts would result in strong mucoadhesion [38–40]. Overall, the nanoliposomes with LCS at a high weight ratio (2 w/w) exhibited both enhanced retention and efficient penetration of the protein, and was selected as the optimal formulation for further evaluation.

### In vitro functional assay of NLp@IFN-λ

**Fig. 4.**
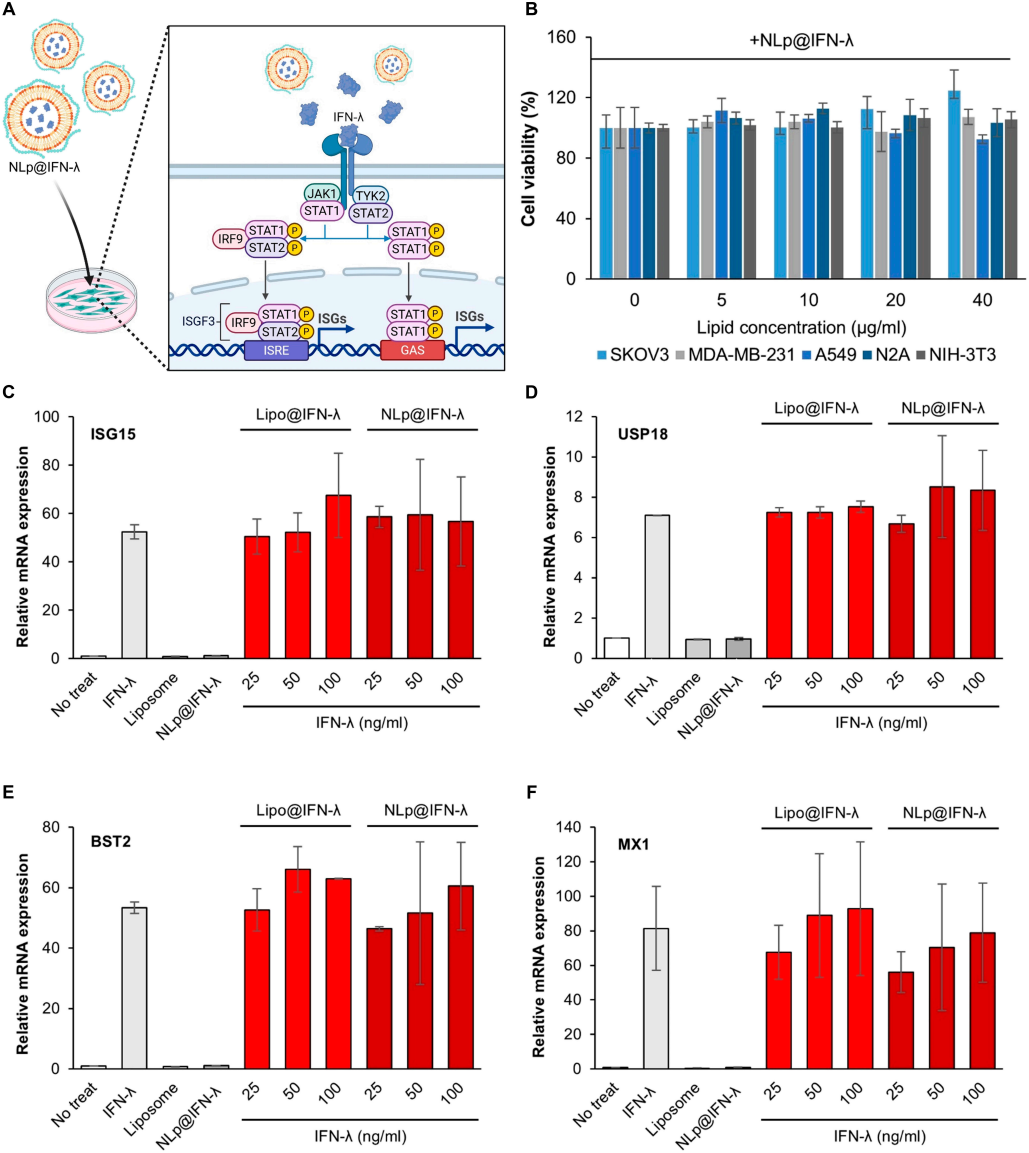
(A) Schematic representing the signal pathway of IFN-λ upon treatment of NLp@IFN-λ, leading to the activation of the JAK/STAT1 pathway and up-regulation of ISG expression. (B) Cytotoxicity of NLp@IFN-λ in various cell lines (*n* = 3). (C to F) ISG responses upon treatment of NLp@IFN-λ at various doses to A549 cells (*n* = 3), by measuring mRNA expression levels of ISG15 (C), USP18 (D), BST2 (E), and MX1 (F). Lipo@IFN-λ (liposomes without CS) was used as the control for comparison (*n* = 3).

We examined the functional activity of the nanoliposomes delivering IFN-λ by treating cells in vitro (Fig. 4A). We first assessed the cytotoxicity of NLp@IFN-λ by treating 5 different cell lines (SKOV3, MDA-MB-231, A549, N2A, and NIH-3T3), which resulted in ~100% viability for up to a lipid concentration of 10 μg/ml, and >92.5% viability at higher levels of treatment (Fig. 4B). To check whether the formulation process of the nanoliposomes affected the biological activity of IFN-λ, we retrieved IFN-λ from NLp@IFN-λ after preparation by disrupting the liposomes and treating A549 and HEK293T cells for quantifying expression levels of 4 different ISGs. In A549 cells, which express the receptor for IFN-λ, significant up-regulation of mRNA expression for all 4 ISGs (ISG15, USP18, BST2, and MX1) was shown for NLp@IFN-λ when treated at IFN-λ concentrations of 10 to 50 ng/ml, which were equivalent to free IFN-λ (Fig. S3). In contrast, treatment of HEK293T cells, which lack the receptor for IFN-λ, with NLp@IFN-λ did not increase the mRNA expression levels of any of the ISGs. These results confirm that the formulation process of the nanoliposomes did not affect the function of IFN-λ, which should activate the Janus kinase (JAK)/signal transducer and activator of transcription 1 (STAT1) signaling pathway and expression of the ISG genes. We observed that cells treated with NLp@IFN-λ did not exhibit any noticeable internalization of IFN-λ (Fig. S4), suggesting that the NLp@IFN-λ remained outside of the cells, which was more suitable for the release of IFN-λ and binding to the IFN-λ receptor on the surface of epithelial cells.

To assess the biological activity of IFN-λ upon delivery by the nanoliposomes, A549 cells were treated with NLp@IFN-λ at various doses and ISG mRNA levels were measured. Treatment with NLp@IFN-λ at concentrations of 25 to 100 ng/ml induced a significant up-regulation of the 4 different ISGs that were over 56.7-, 8.3-, 60.5-, and 78.9-fold (vs. no treatment) for ISG15, USP18, BST2, and MX1, respectively (Fig. 4C to F). The extent of up-regulation was not significantly different between NLp@IFN-λ and liposomes without CS (Lipo@IFN-λ), and was comparable to results from treating free IFN-λ. These results demonstrate that the nanoliposomes could encapsulate and release IFN-λ while preserving its bioactivity. On the other hand, treatment with blank anionic liposomes or blank liposomes with CS did not induce ISG expression, confirming that the response was specifically mediated by IFN-λ signaling and not by nonspecific stimulation from the composition of the nanoliposomes. Taken together, these results demonstrate that NLp@IFN-λ provides a robust delivery platform that can release the therapeutic protein in a sustained manner without the loss of its bioactivity, suggesting its potential as a nasal formulation.

### In vivo delivery and efficacy of NLp@IFN-λ

**Fig. 5.**
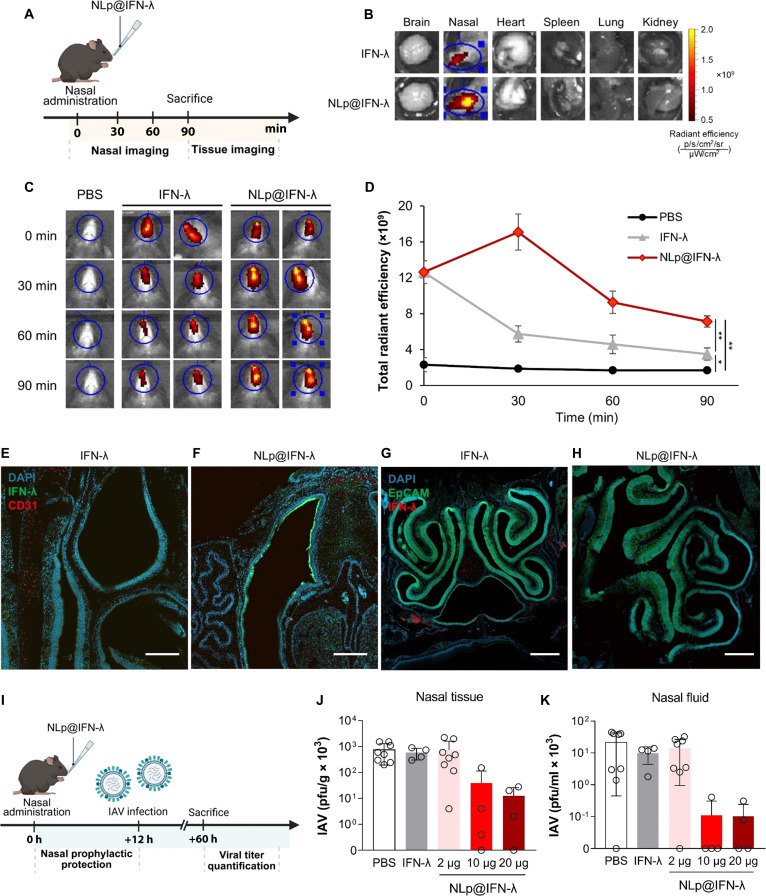
In vivo delivery and efficacy of NLp@IFN-λ. (A) Schematic for nasal delivery of NLp@IFN-λ into mice and fluorescence imaging of tissues. (B) Total radiant efficiency of IFN-λ in major tissues harvested at 90 min after administration. (C) Imaging of mice including site of injection and surrounding nasal tissues, after intranasal administration of NLp@IFN-λ, and detection of IFN-λ fluorescence at various time points. IFN-λ was labeled with AF750 for fluorescence imaging in (B) and (C). (D) Quantification of total radiant efficiencies of (C) (*n* = 3, *P < 0.05, **P < 0.01). (E and F) Confocal images of respiratory epithelium (E and F, scale bar: 200 μm) and olfactory epithelium (G and H, scale bar: 500 μm) after intranasal administration of NLp@IFN-λ or free IFN-λ as the control. (I) Schematic of the timeline for efficacy studies. Mice were injected with NLp@IFN-λ by nasal administration, followed by infection with IAV at 12 h, and sacrificed at 60 h postinfection. (J and K) Quantification of viral titers in nasal tissues (J) and nasal fluids (K) of mice at 2 days postinfection by the standard plaque assay (*n* = 4 to 8, bars represent mean ± SD).

We next evaluated the nanoliposomes delivering IFN-λ as a nasal formulation for treatment in vivo. NLp@IFN-λ was intranasally administered into mice, followed by imaging the nasal tissue and other organs (Fig. 5A). Results showed that the signals of the fluorescence-labeled IFN-λ were localized in the nasal tissue, while other tissues such as heart, lung, spleen, kidney, and brain did not exhibit any signal (Fig. 5B). Quantification of the total radiant efficiency of IFN-λ in the nasal tissue was 2.1-fold higher for the NLp@IFN-λ treatment group compared to the free IFN-λ group at 90 min posttreatment (Fig. S5). Longitudinal monitoring of the fluorescence signal of IFN-λ in the nasal tissues revealed accumulation and prolonged retention for the NLp@IFN-λ treatment group for up to 90 min posttreatment, whereas free IFN-λ showed rapid clearance (Fig. 5C). Quantification of total radiant efficiency showed that the values increased to 2.0-fold for NLp@IFN-λ at 90 min posttreatment, compared to free IFN-λ (Fig. 5D). Immunohistochemistry of the respiratory epithelium revealed that delivery by NLp@IFN-λ resulted in substantial localization of IFN-λ along the respiratory epithelium, while free IFN-λ did not (Fig. 5E and F). On the other hand, the olfactory epithelium did not show any significant signal for both the NLp@IFN-λ and free IFN-λ treatment groups (Fig. 5G and H). These results suggest that the nanoliposomes provide an effective platform for delivery via the intranasal route that can enhance penetration through the nasal mucosa while minimizing delivery to the olfactory epithelium, which can cause potential side effects in the central nervous system.

To assess the antiviral efficacy in vivo, a challenge model of IAV infection in mice was generated. NLp@IFN-λ was intranasally administered, followed by infection with IAV after 12 h. At 48 h postinfection, viral titers were quantified in the different respiratory compartments (Fig. 5I). In the case of nasal tissues, NLp@IFN-λ resulted in 94.8% and 98.3% reduction in viral titers compared to the PBS control for the 10- and 20-μg treatment, respectively (Fig. 5J). The results for NLp@IFN-λ showed a 93.3% to 97.9% decrease in viral titers compared to free IFN-λ treatment. In the nasal fluids, NLp@IFN-λ treatment resulted in a ~99.5% decrease compared to the PBS control for 10 and 20 μg, which showed a ~99.0% reduction compared to free IFN-λ (Fig. 5K). These results can be due to the enhanced retention of the protein in the nasal tissues by NLp@IFN-λ that interacts with the nasal epithelium, while free IFN-λ diffuses rapidly into the surrounding respiratory tissues and fluid, to induce the antiviral responses. Compartments of the lower respiratory tract, including the lung tissue and BAL fluid, also showed potent effects when treated with NLp@IFN-λ at 10 and 20 μg, which can be due to the high dose of treatment (Fig. S6). These findings demonstrate the balance between mucoadhesive and mucus-penetrating properties of NLp@IFN-λ, which promotes sustained and local release of the protein in the upper respiratory tract. The nanoliposomes can also provide protection of IFN-λ from proteolytic degradation in the nasal cavity as well as the lung tissues, promoting efficient delivery and efficacy. Collectively, these results demonstrate that NLp@IFN-λ could effectively prevent early-stage viral replication in the upper respiratory tract, leading to localized antiviral responses. Furthermore, treatment of NLp@IFN-λ can also prevent transmission of the virus from the upper to the lower respiratory tract that can result in systemic prophylactic effects.

## Conclusion

In this study, we developed an intranasal nanoliposome platform for the delivery of IFN-λ as an antiviral. Our nanoliposomes (NLp@IFN-λ) were optimal to achieve both mucoadhesion and controlled release, leading to efficient diffusion through the nasal mucus layer, while preserving the bioactivity of IFN-λ. The nanoliposomes could encapsulate IFN-λ with high efficiency, allowing sustained release and mucus penetration, which can potentially lead to prolonged antiviral efficacy. Treatment of NLp@IFN-λ to cells in vitro could induce IFN-stimulated gene expression without causing cytotoxicity, which also confirmed that the bioactivity of IFN-λ was maintained. Furthermore, in vivo delivery via an intranasal route demonstrated that NLp@IFN-λ enhanced the retention and localization of the protein in the nasal cavity and resulted in significant prophylactic antiviral effects against IAV. While NLp@IFN-λ showed more potent localized antiviral responses in the upper respiratory tract, higher-dose treatment could expand its efficacy to the lower respiratory tract. The current study highlights the therapeutic potential of NLp@IFN-λ as an intranasal delivery platform, as well as a noninvasive antiviral formulation for prophylaxis against various emerging respiratory pathogens.

## Data Availability

The data that support the findings of this study are available from the corresponding authors upon reasonable request.
