# Supplementary material for: Intranasal Nanoliposomes Delivering Interferon Lambda with Enhanced Mucosal Retention as an Antiviral
Source: Biomater Res. 2025 Nov 21;29:0287. doi: 10.34133/bmr.0287 (PMC12636035; doi:10.34133/bmr.0287)
Supplement: Supplementary 1 — Table S1 Figs. S1 to S6 [file bmr.0287.f1.docx]

SUPPLEMENTARY MATERIALS

***Table S1.*** *Sequences oligonucleotides for qPCR primers.*

| Type of oligonucleotide | Sequences of oligonucleotides |
| --- | --- |
| Isg15_Fwd | TCCTGCTGGTGGTGGACAA |
| Isg15_Rev | TTGTTATTCCTCACCAGGATGCT |
| Mx1_Fwd | CCGTGACGGATATGGTCCGGC |
| Mx1_Rev | CTGGAAGTGGAGGCGGATCAGC |
| Usp18_Fwd | TGGACAGACCTGCTGCCTTAA |
| Usp18_Rev | CTGTCCTGCATCTTCTCCAGC |
| Bst2_Fwd | TCTCCTGCAACAAGAGCTGAC |
| Bst2_Rev | TCTCTGCATCCAGGGAAGCCA |
| GAPDH_Fwd | CAATGCCTCCTGCACCACCA |
| GAPDH_Rev | GATGTTCTGGAGAGCCCCGC |


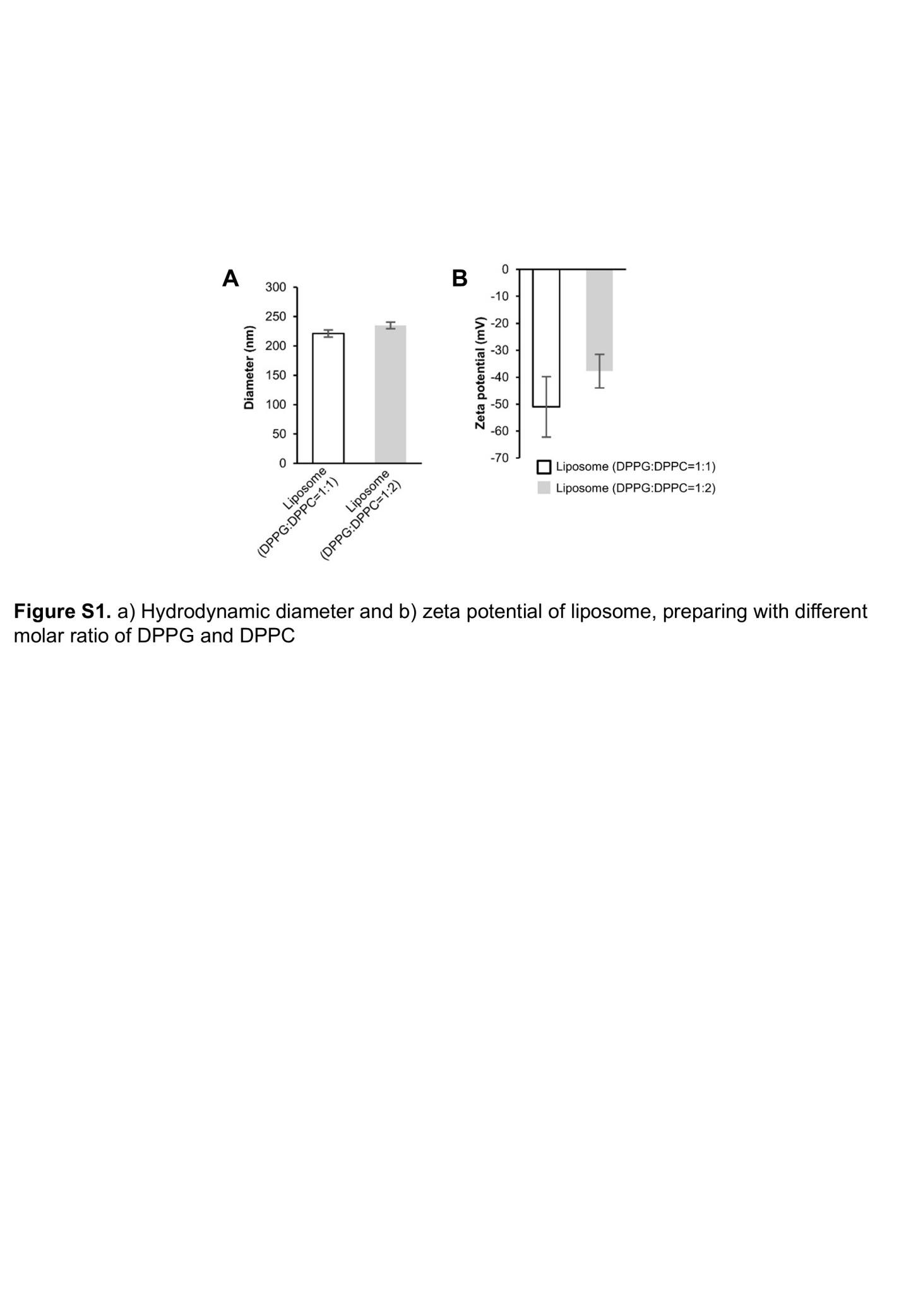


***Fig. S1.*** *(A) Hydrodynamic diameter and (B) zeta potential of liposome, preparing with different molar ratio of DPPG and DPPC*


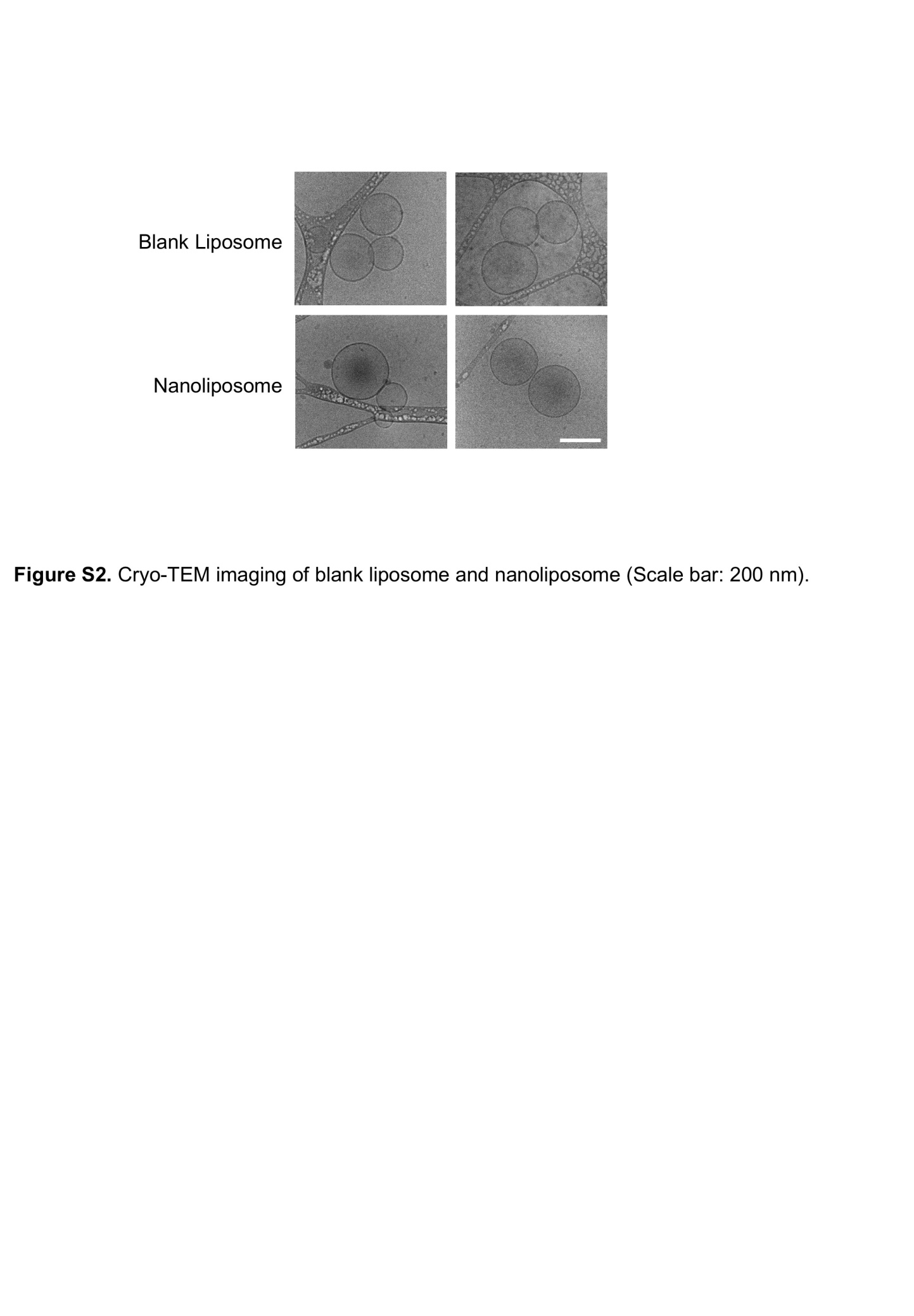


***Fig. S2.*** *Cryo-TEM imaging of blank liposome and nanoliposome (Scale bar: 200 nm).*


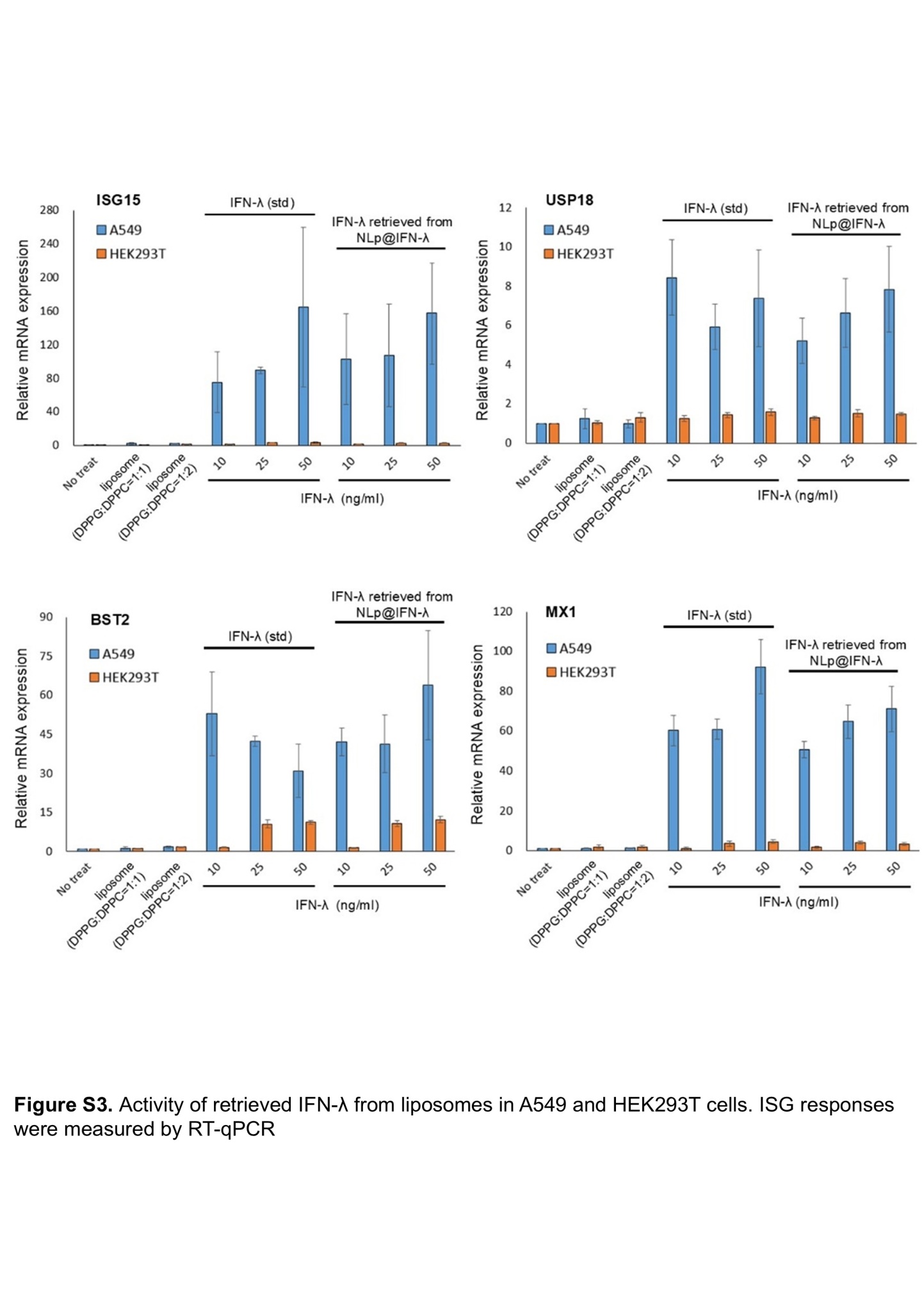


***Fig. S3.*** *Activity of retrieved IFN-λ from liposomes in A549 and HEK293T cells. ISG responses were measured by RT-qPCR.*


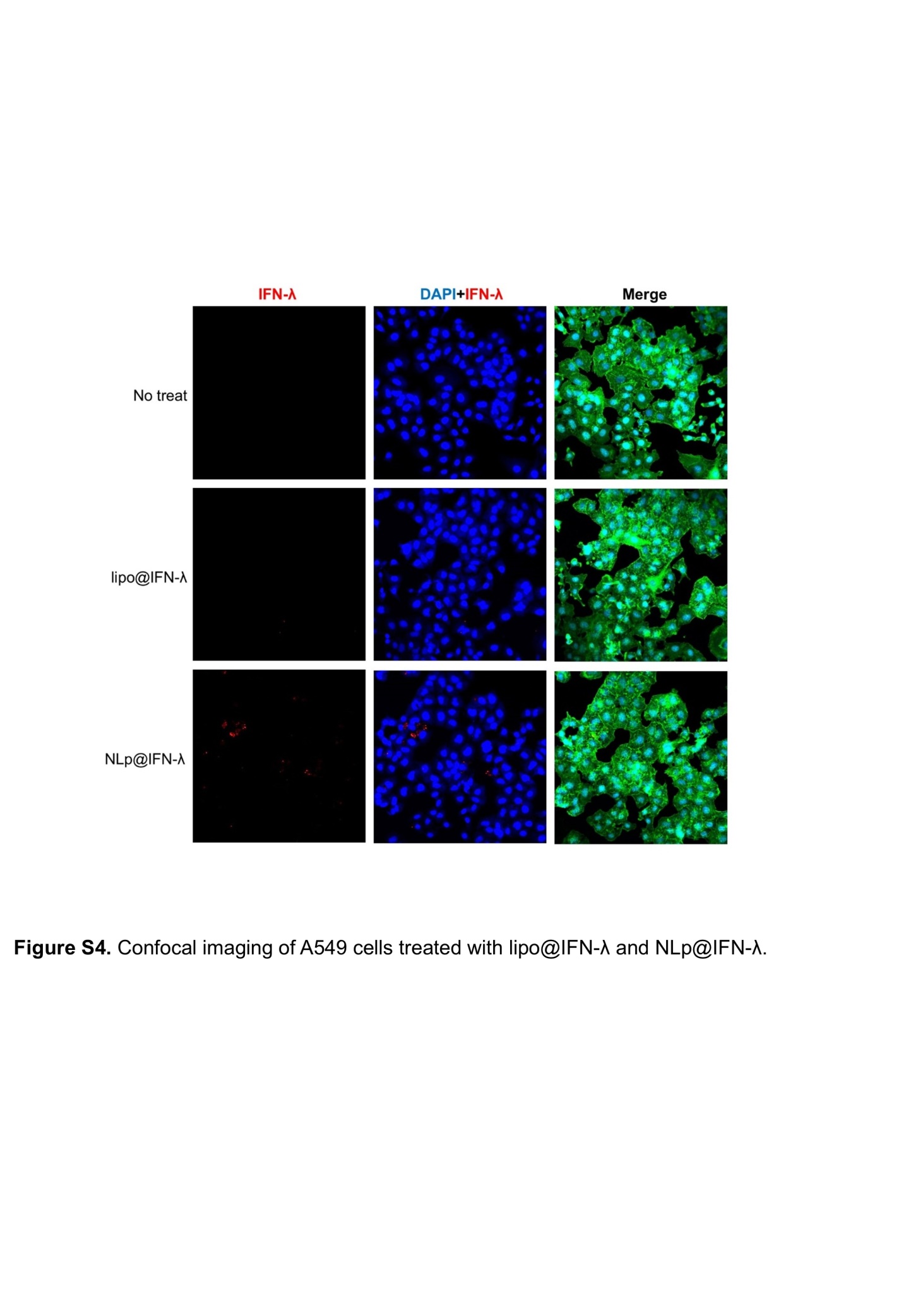


***Fig. S4.*** *Confocal imaging of A549 cells treated with lipo@IFN-λ and NLp@IFN-λ.*


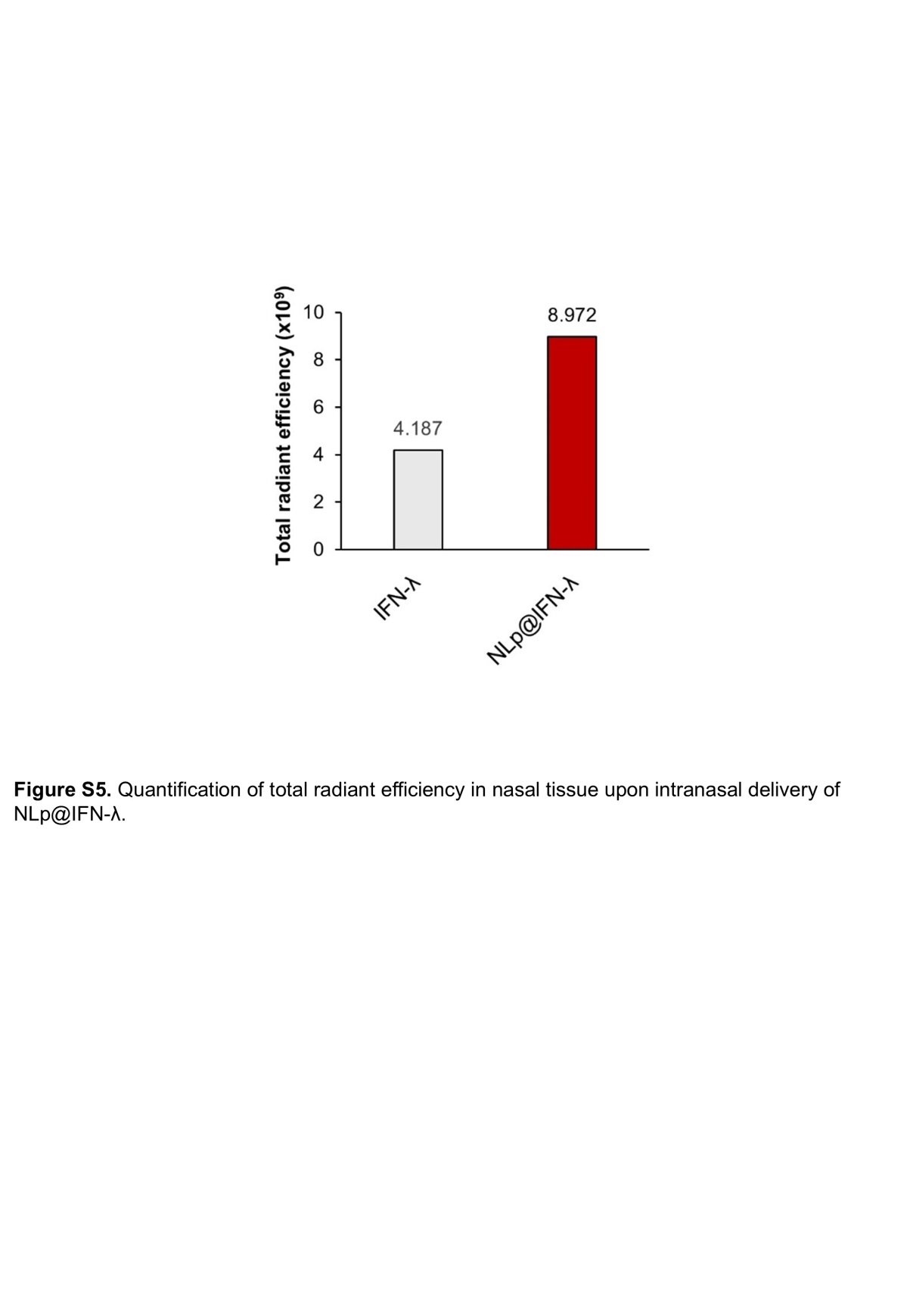


***Fig. S5.*** *Quantification of total radiant efficiency in nasal tissue upon intranasal delivery of NLp@IFN-λ.*


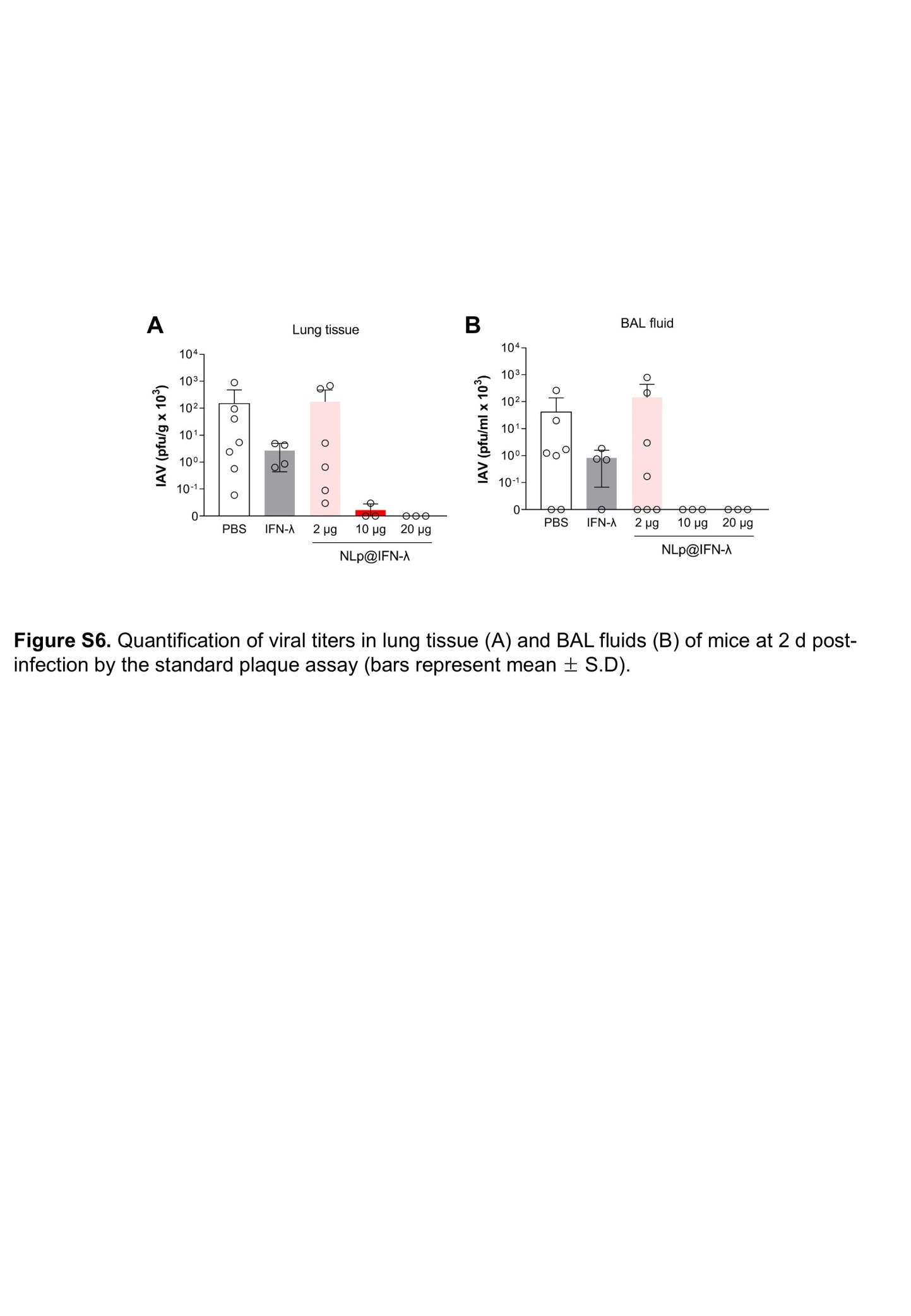


***Fig. S6.*** *Quantification of viral titers in lung tissue (A) and BAL fluids (B) of mice at 2 d post-infection by the standard plaque assay (bars represent mean ± S.D).*
